# Dental visits among adolescents in Brazil: descriptive analysis of the National School Health Survey, 2019

**DOI:** 10.1590/S2237-96222025v34e20240344.en

**Published:** 2025-06-13

**Authors:** Hellen Monique da Motta, Nathalia Ribeiro Jorge da Silva-Garcia, Letícia Regina Morello Sartori, Maria Beatriz Junqueira de Camargo, Sarah Arangurem Karam

**Affiliations:** 1 Universidade Federal de Pelotas, Faculdade de Odontologia, Curso de Odontologia, Pelotas, RS, Brazil; 2 Universidade Federal de Pelotas, Programa de Pós-Graduação em Odontologia, Pelotas, RS, Brazil; 3 Universidade Católica de Pelotas, Mestrado Profissional em Saúde no Ciclo Vital, Pelotas, RS, Brazil

**Keywords:** Dental Care, Adolescent, Access to Primary Care, Health Inequality Indicators, Unified Health System, Atención Odontológica, Adolescente, Acceso a Atención Primaria, Indicadores de Desigualdad en Salud, Sistema Único de Salud

## Abstract

**Objective:**

To describe the prevalence of dental visits among adolescents participating in the 2019 National School Health Survey (PeNSE).

**Methods:**

A descriptive cross-sectional study analyzing dental visits in the last year considering sex, race/skin color, maternal education and dental pain. Only PeNSE 2019 participants between ages 13 and 15 years were included. Absolute and relative frequencies were verified using Pearson’s Chi-square test, with a 5% confidence level (p-value<0.05) and the respective 95% confidence intervals (95%CI). As a secondary analysis, data on dental coverage in primary health care were collected and reported according to Brazilian regions.

**Results:**

The prevalence of visits to the dentist in the last year was 66.7%; a higher prevalence was noted for adolescents who declared themselves white compared to black adolescents (71.8% versus 61.9%), for females (68.4%; 95%CI 67.27; 69.49), among students with toothache (69.4%; 95%CI 67.61; 71.10) and with mothers with higher education (77.9%; 95%CI 76.56; 79.18). Federative units with high dental coverage reported lower use of services, while those with lower coverage reported a higher proportion of visits to the dentist.

**Conclusion:**

Among adolescents aged 13 to 15 years, a 66.7% prevalence of visits to the dentist in the last year was observed; higher prevalences of visits to the dentist were found among female adolescents, self-declared white, with mothers with higher education and with self-reported toothache. Furthermore, there was a lower prevalence of visits to the dentist in places with greater dental coverage.

## Resumen

**Objetivo:**

Describir la prevalencia de visitas odontológicas entre adolescentes participantes en la Encuesta Nacional de Salud Escolar (PeNSE) de 2019.

**Métodos:**

Estudio descriptivo transversal que analizó las visitas odontológicas realizadas durante el último año, considerando sexo, raza/color de piel, educación materna y dolor dental. Solo se incluyeron participantes de la PeNSE 2019 de entre 13 y 15 años. Las frecuencias absolutas y relativas se verificaron mediante la prueba de Chi-cuadrado de Pearson, con un nivel de confianza del 5% (valor-p<0,05) y sus respectivos intervalos de confianza del 95% (IC95%). Como análisis secundario, se recopilaron datos sobre la cobertura odontológica en atención primaria de salud, que se reportaron según las regiones brasileñas.

**Resultados:**

La prevalencia de visitas al dentista durante el último año fue del 66,7%; Se observó una mayor prevalencia en adolescentes que se declararon blancos en comparación con los adolescentes negros (71,8% versus 61,9%), en mujeres (68,4%; IC95% 67,27; 69,49), entre estudiantes con dolor de muelas (69,4%; IC95% 67,61; 71,10) y con madres con educación superior (77,9%; IC95% 76,56; 79,18). Las unidades federativas con alta cobertura dental reportaron un menor uso de los servicios, mientras que aquellas con menor cobertura reportaron una mayor proporción de visitas al dentista.

**Conclusión:**

Entre los adolescentes de 13 a 15 años, se observó una prevalencia del 66,7% de visitas al dentista en el último año; se encontraron mayores prevalencias de visitas al dentista entre las adolescentes mujeres, autodeclaradas blancas, con madres con educación superior y con dolor de muelas autoreportado. Además, hubo una menor prevalencia de visitas al dentista en lugares con mayor cobertura dental.

**Ethical aspects**

This research used public domain and anonymized databases.

## Introduction

In 2022, approximately one in seven Brazilians were adolescents between the ages of 10 and 19 (1). Adolescence is a period of fundamental importance in human development, in which different physical, psychological and emotional changes occur (2). A transitional period between childhood and adulthood, it is during adolescence that new health behaviors are adopted, and, once established, they have a direct impact on the adult life of individuals (2-3). In particular, new health behaviors emerge, related to diet, physical activity, sexual activity and substance use, such as alcohol and drugs (2-3). Many of these behaviors can contribute to the establishment or worsening of oral health problems – such as tooth decay, gingivitis, periodontal disease and malocclusions, which can directly interfere with function and aesthetics, social development and school performance, in addition to contributing to school absenteeism (4-7). As a result of these conditions, dental pain and aesthetic problems also cause significant harm to the quality of life related to oral health, especially in the areas of social and emotional well-being (8). 

During adolescence, significant changes occur in behavioral characteristics in terms of oral health, closely related to changes in social influences and growing individual autonomy and independence (2-3,9). For example, a British cohort study found a decreasing use of dental services in the last year between childhood and late adolescence, with the decline being most pronounced between the ages of 10 and 17 (10). Given the different patterns of use of health services between different periods in adolescence, the World Health Organization recommends monitoring the health of students through surveillance of behavioral risk factors and protective factors in this age group (11). From this perspective, the Ministry of Health has encouraged the monitoring of student’s health by municipalities, seeking to plan and implement actions that promote health and that can last throughout the life cycle (12).

Globally and in Brazil, although young people aged 10 to 19 are not in the age groups with the highest peak prevalence and incidence of oral health problems, they represent a period in which there is a progressive escalation in the rates of dental caries and periodontal disease until adulthood (9,13). Therefore, this period is a relevant window of opportunity for interventions that improve the oral health of individuals throughout the life cycle. In Brazil, preliminary data from the 2022 National Oral Health Study revealed that the number of young people aged 15 to 19 years free of cavities increased from 29.8% in 2010 to 33% in 2022 (14). However, the untreated disease component within the index increased (40% in 2010 *versus* 52.9% in 2022), which suggests a considerable worsening in the oral health of Brazilian adolescents and a greater need for dental treatment (14). Inline with these findings, a study carried out with data from the National School Health Survey (PeNSE) observed a significant increase in the prevalence of self-reported dental pain, from 18.8% in 2009 to 23.7% in 2015 (4).

Considering the significant burden of oral conditions in adolescents, it is essential to understand the sociodemographic factors associated with their use of dental services. Studies show that young females (15-16), whose mothers have higher education and higher family income are more likely to have gone to the dentist than those with worse socioeconomic status (16-17). Furthermore, racial and macro-regional inequities in the use of health services are present; adolescents aged 13 to 17 and of non-white skin color are less likely to have sought dental services in the last year (14) and adolescents (15 to 19 years old) from the North, Northeast and Midwest regions are more likely to have never visited the dentist (18); despite this, both groups use proportionally more dental services of the Unified Health System (SUS) (16,18). However, little is known about how these socioeconomic factors impact the use of dental services by adolescents aged 13 to 15 years. Thus, the objective of the present study was to describe the prevalence of visits to the dentist in the last year among adolescents aged 13 to 15 years participating in PeNSE 2019. 

## Methods

### Study design

This descriptive cross-sectional study used secondary data from PeNSE in 2019, which contains a representative sample of the Brazilian school population aged 13 to 17. 

### Context and participants

PeNSE is a study carried out by the Brazilian Institute of Geography and Statistics in association with the Ministry of Health and the Ministry of Education. Its four editions were carried out in 2009, 2012, 2015 and 2019 (19-20). The 2019 edition was held in public and private schools, with national coverage and data available for Brazil as a whole, Major Regions, Federation Units and Capital Cities (19). Students included in the research should be between ages 13 and 17 years and should be regularly enrolled and attending school, considering the 6th to 9th grade of elementary school and the 1st to 3rd year of high school (19). To compose the sample, a two-stage cluster sampling was used, with schools being the first stage and classes being the second stage (19). Additional information about PeNSE 2019 can be found in a previous publication (16). 

### Eligibility criteria

In the present study, only data from adolescents aged 13 to 15 years that presented valid information for the dependent variable evaluated were included. The age range restriction is related to the expansion of the scope of the sample of students in the 2019 edition of PeNSE, compared to previous editions, now including representation by age groups from 13 to 15 years and from 16 to 17 years (19). According to estimates, the majority of adolescents attending school in Brazil are between the ages of 13 and 15, which correspond to the sample of this study (19).

### Variables of interest

The dependent variable was visits to the dentist in the last year, measured by the question “In the last twelve months, how many times did you go to the dentist?” This question could be answered with the following answer options: “Never in the last 12 months”; “Once”; “Twice” or “3 or more times”. For statistical analysis purposes, the answers were dichotomized into “No” and “Yes”, with visits to the dentist in the last year being included in the “yes” category, regardless of the number of times.

The following independent variables were used: sex (Male and Female), race/skin color (White, Black, Brown, Asian and Indigenous), Brazilian region (North, Northeast, Midwest, Southeast and South), maternal education (No education/Incomplete Elementary Education, Complete Elementary Education, Complete High School and Complete Higher Education), and toothache in the last six months (No and Yes). 

### Statistical analysis

Data analysis was performed using Stata 18.0 statistical software (Stata Corp, College Station, Texas, United States of America). The absolute and relative frequencies of the dependent variable, visits to the dentist in the last year, in relation to the independent variables, were verified using Pearson’s chi-square test. A confidence level of 5% (p-value<0.05) and the respective 95% confidence intervals (95%CI) were established The svy command for outlining purposes was used.

Furthermore, a complementary ecological analysis was performed using secondary data on oral health coverage. These data were collected by a researcher in March 2024, through the Information System of the Secretariat of Primary Health Care, in the primary health care indicator panels related to oral health, available online at sisaps.saude.gov.br/painelsaps/saude-bucal. The relationship between primary care oral health coverage by the SUS as a contextual variable, as well as visits to the dentist in the last year by the students, was presented graphically. Secondary data were collected by federation unit, filtered for the year 2018, the year closest to the results of PeNSE 2019.

## Results

PeNSE 2019 estimated 124,898 students between ages 13 and 17 years. Of these, students aged 13 to 15 made up around 66% of the sample, which is the proportion included in this study. 

Among adolescents aged 13 to 15, 50.8% were female, 39.8% lived in the southeast region, 43.2% self-declared as brown, and 31.2% of participants were in the 9th year of elementary school (Table 1). 21.4% reported having felt toothache in the last six months (Table 1). Furthermore, a prevalence of 66.7% in dental services use among adolescents in the last 12 months can be observed (Table 1). 

**Table 1:** Proportion (%) and 95% confidence interval (95%CI) of the sample, according to sociodemographic characteristics, use of dental services and dental pain in Brazilian students aged 13 to 15 years, data from the National School Health Survey. Brazil, 2019 (n=82,389)

	%	95%CI
Sex		
Male	49.2	48.27; 50.04
Female	50.8	49.96; 51.73
**Race/skin color**		
White	36.8	35.72; 37.82
Black	12.8	12.15; 13.49
Brown	43.2	42.19; 44.28
Yellow (Asian)	3.8	3.47; 4.08
Indigenous	3.4	3.14; 3.76
**Brazilian region**		
North	10.3	9.13; 11.57
Northeast	28.1	26.34; 29.94
Midwest	8.3	7.65; 9.05
Southeast	39.8	37.38; 42.28
South	13.5	12.35; 14.70
**School year**		
6th grade of elementary school	0.2	0.17; 0.32
7th grade of elementary school	11.1	9.79; 12.58
8th grade of elementary school	29.9	26.99; 32.91
9th grade of elementary school	31.2	27.76; 34.77
1st year of high school	23.4	20.54; 26.57
2nd year of high school	4.1	3.27; 5.12
3rd year of high school	0.1	0.04; 0.17
**Maternal education**		
No education/incomplete primary education	26.8	25.78; 27.94
Completed elementary education	17.1	16.38; 17.80
Completed high school	32.4	31.56;33.21
Completed higher education	23.7	22.80; 24.62
**Toothache in the last 6 months**		
No	78.6	77.87; 79.31
Yes	21.4	20.69; 22.13
**Visit to the dentist in the last year**		
No	33.3	32.34; 34.21
Yes	66.7	65.79; 67.66
**Frequency of visits to the dentist in the last year**		
Once	22.0	21.31; 22.82
Twice	22.6	21.82; 23.37
Three times or more	55.4	54.36; 56.35

Table 2 also notes that female adolescents were more likely to report the use of dental services, being represented by 68.4% (95%CI 67.27; 69.49), with a difference of a little over 3 percentage points when compared to the male sex, represented by 65% (95%CI 63.83; 66.19). Adolescents who self-declared as white had a higher prevalence of visits to the dentist in the last year (71.8%; 95%CI 70.54; 72.92), a difference of 10 percentage points in relation to adolescents who self-declared as black (61.9%; 95%CI 59.76; 63.93). It was also noted that adolescents whose mothers had not attended school or had incomplete elementary education had a prevalence of visits to the dentist of 61.6% (95%CI 59.89; 63.24), while adolescents with mothers who completed higher education had a prevalence of 77.9% (95%CI 76.56; 79.18) for visits to the dentist in the last year (table 2). Additionally, it was noted that 69.4% (95%CI 67.61; 71.10) of the adolescents who confirmed having had toothache in the last six months had visited a dentist in the last year (Table 2).

**Table 2:** Proportion (%) and 95% confidence interval (95%CI) of visits to the dentist in the last year, according to sociodemographic characteristics and dental pain in Brazilian students aged 13 to 15, data from the National School Health Survey. Brazil, 2019 (n=82,389)

	Visit to the dentist in the last year		
	No (95%CI)	Yes (95%CI)	p-value
Sex			<0.001
Male	35.0 (33.81; 36.17)	65.0 (63.83; 66.19)	
Female	31.6 (30.51; 32.73)	68.4 (67.27; 69.49)	
**Race/skin color**			<0.001
White	28.3 (27.08; 29.46)	71.7 (70.54; 72.92)	
Black	38.1 (36.07; 40.24)	61.9 (59.76; 63.93)	
Brown	35.8 (34.43; 37.20)	64.2 (62.80; 65.57)	
Yellow (Asian)	36.4 (32.66; 40.25)	63.6 (59.75; 67.34)	
Indigenous	33.6 (30.21; 37.25)	66.4 (62.75; 69.79)	
**Maternal education**			<0.001
No education/incomplete primary education	38.4 (36.76; 40.11)	61.6 (59.89; 63.24)	
Completed elementary education	34.9 (32.77; 37.20)	65.1 (62.80; 67.23)	
Completed high school	30.5 (29.24; 31.88)	69.5 (68.12; 70.76)	
Completed higher education	22.1 (20.82; 23.44)	77.9 (76.56; 79.18)	
**Toothache in the last 6 months**			<0.001
No	34.5 (33.37; 35.61)	65.5 (64.39; 66.63)	
Yes	30.6 (28.90; 32.39)	69.4 (67.61; 71.10)	

**Figure 1:**
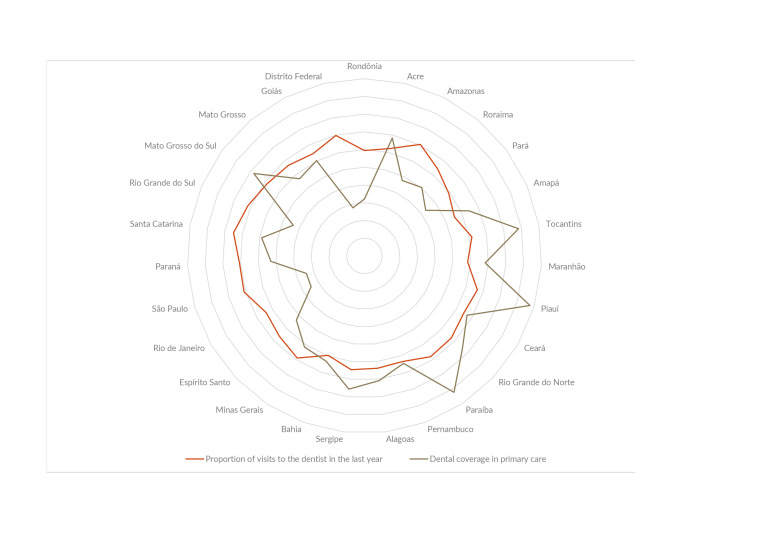
Overlap of the proportion of visits to the dentist in the last year with data from the 2019 National School Health Survey, in relation to primary care dental coverage by Federative Unit with data from the Information System of the Secretariat of Primary Health Care in Brazil, 2018

Figure 1 illustrates the overlap of dental coverage in primary care and the reported proportion of visits to the dentist in the last year among students by Federation Unit. The greatest coverage was observed for the state of Piauí, with 97.5% of the population assisted, followed by Paraíba, with 92.1% and Tocantins with 88.3% (Figure 1). In contrast, the Federal District had a coverage of only 27.9% for the population (Figure 1). In terms of visits to the dentist in the last year by state, the state of Santa Catarina reported 75% of adolescents, followed by Rio Grande do Sul (71.6%), São Paulo (70.9%) and Paraná (70.6%); the lowest proportion was observed in the state of Amapá, with 55.3%. Thus, it can be highlighted that federative units with high coverage, such as those in the North and Northeast regions, had an overlap with a lower report of use of services by adolescents, while federative units with lower coverage had a higher proportion of students going to the dentist in the last year. 

## Discussion

Among the findings of this study, the high prevalence of use of dental services among adolescents aged 13 to 15 participating in PeNSE 2019 is worth noting. Furthermore, there was a greater report of use of dental services by self-reported white race/skin color female adolescents with mothers with higher levels of education and who had experienced toothache in the last six months compared to their counterparts. Additionally, the inverse graphic overlap between oral health coverage and the proportion of visits to the dentist in the last year by federative units also deserves mention.

Although the prevalence of use of dental services in the last year can be considered high compared to other middle-income countries (21-22), there was a reduction compared to data from the previous edition of PeNSE, in 2015 (4.19). This reduction not only suggests a lower demand for dental services, but may also result in the emergence of new oral health problems or the progression of pre-existing oral diseases (23). Furthermore, it was observed that the greater report of dental pain in the last six months was associated with a greater use of dental services in the last year, which reinforces an already observed pattern of use of oral health services mainly for curative purposes (16-17).

Corroborating the findings of this study, a survey conducted with data from PeNSE in 2015 had observed a greater reporting of the use of health services by female students (16). Furthermore, national data showed that women tend to seek health services more (24). This greater use of service can be attributed to the concern with health issues and care, increasing the possibilities of diagnosis and prevention. The observation of a significant difference in the frequency of dental consultations between white and non-white young people reinforces that this study also reiterates the impact of racial inequities in the use of health services. It has been found that black individuals are more likely to undergo irregular dental treatment or even never have consulted the dentist (25). This discrepancy was also found in a study with Brazilian adolescent students, which observed that black and brown young people suffer more from dental pain compared to white people (26). From an early age, black people have to deal with worse living conditions, which impact the access to and use of health services, in addition to dealing with structural, institutional and interpersonal racism that quantitatively impacts the health care receive (25,27).

Furthermore, it is noted that visits to the dentist in the last year tend to increase as the level of maternal education increases, with a difference in the prevalence of visits to the dentist of 16 percentage points between those with mothers with less education and those with more education. This may reflect less knowledge about oral health care, difficulties in accessing information about health services and, mainly, the presence of dentists in basic health units (28). The issue of family income must be noted, since a higher level of education is associated with greater economic power, so family groups with greater purchasing power tend to access more health services, most often in private practice (29-30). A lower frequency of visits to the dentist in the group of adolescents with less educated mothers may be related to the opening hours of public health services, as they normally coincide with school hours or even, if the student works part-time, with their work hours or also those of their parents (4.31).

Two main factors could explain he inverse relationship between the proportion of oral health coverage in primary care and the proportion of visits to the dentist in the last year, observed in the present study. More remote regions of the country, mainly represented by the northern region, have difficulties in terms of physical spaces, movement of teams and retention of professionals, but their population still has public health services as their main reference (32-33). However, since states with high primary care coverage are also those with a higher concentration of income, this reinforces the idea that consultations are mostly carried out in private services or through health insurance plans, or it may reflect low trust in the services provided by the Unified Health System (18.34). 

Among the strengths of this study, we can mention the use of a representative sample for the universe of Brazilian students between ages 13 and 15 years, which guarantees the generalization of our findings. Also, the use of data from the latest edition of PeNSE is reinforced, which updates the literature already available on the topic and the specific focus on the stratum of young people aged 13 to 15. However, some limitations should be highlighted. It is important to note that more than half of adolescents aged 13 to 15 who visited the dentist in the last 12 months claimed to have gone three or more times. As the questionnaire applied to the students did not rule out visiting the dentist for orthodontic treatment, a very common procedure at the age analyzed in this study, this prevalence may have been influenced by adherence to and continuity of this type of treatment. (23). Therefore, a relevant limitation of this research is the fact that the data do not allow distinguishing between preventive and curative care, making it difficult to conduct a more detailed analysis of the reason for using dental services in this population. Furthermore, as only adolescents who are duly enrolled and attending school are eligible for PeNSE, the results found here are not generalizable to young people who are out of school and, consequently, more vulnerable. 

In conclusion, the findings of this study indicate that the use of dental services by adolescents aged 13 to 15 in Brazil may be associated with socioeconomic and demographic inequalities, highlighting inequities in access to the service. Furthermore, the inverse relationship observed between primary care dental coverage and visits to the dentist suggests the need to investigate possible barriers to the use of these services. These results reinforce the importance of public policies that can guarantee more equitable access to dental care from early adolescence, contributing to the reduction of inequalities in oral health.

## Data Availability

The data used in this article are available at: https://osf.io/swn8y/?view_only=cf392fd83fc54493b836f8c463063f57.
