# Supplementary material for: Dental visits among adolescents in Brazil: descriptive analysis of the National School Health Survey, 2019
Source: Epidemiol Serv Saude. 2025 Jun 13;34:e20240344. doi: 10.1590/S2237-96222025v34e20240344.en (PMC12176439; doi:10.1590/S2237-96222025v34e20240344.en)
Supplement: Translation

# Translation: Consulta Odontológica entre Adolescentes no Brasil: análise descritiva da Pesquisa Nacional da Saúde do Escolar, 2019

**DOI:** 10.1590/S2237-96222025v34e20240344.pt

**Authors:** Hellen Monique da Motta, Nathalia Ribeiro Jorge da Silva-Garcia, Letícia Regina Morello Sartori, Maria Beatriz Junqueira de Camargo, Sarah Arangurem Karam

**Affiliations:** 1 Universidade Federal de Pelotas, Faculdade de Odontologia, Curso de Odontologia, Pelotas, RS, Brasil; 2 Universidade Federal de Pelotas, Programa de Pós-Graduação em Odontologia, Pelotas, RS, Brasil; 3 Universidade Católica de Pelotas, Mestrado Profissional em Saúde no Ciclo Vital, Pelotas, RS, Brasil

**Keywords:** Assistência Odontológica, Adolescente, Acesso à Atenção Primária, Indicadores de Desigualdade em Saúde, Sistema Único de Saúde

## Abstract

**Objetivo:**

Descrever a prevalência de ida ao dentista entre adolescentes participantes da Pesquisa Nacional de Saúde do Escolar (PeNSE) ano de 2019.

**Métodos:**

Estudo com delineamento transversal descritivo que analisou a ida ao dentista no último ano considerando sexo, raça/cor da pele, escolaridade materna e dor dentária. Foram incluídos apenas os participantes da PeNSE 2019 entre 13 e 15 anos. As frequências absolutas e relativas foram verificadas através do teste Qui-quadrado de Pearson, com nível de confiança de 5% (p-valor<0,05) e os respectivos intervalos de confiança de 95% (IC95%). Como análise secundária, foram coletados e relacionados os dados da cobertura odontológica na atenção primária segundo as regiões brasileiras.

**Resultados:**

A prevalência de ida ao dentista no último ano foi de 66,7%; maior prevalência foi notada para adolescentes autodeclarados brancos em comparação aos pretos (71,8% versus 61,9%), para o sexo feminino (68,4%; IC95% 67,27; 69,49), entre os escolares com dor de dente (69,4%; IC95% 67,61; 71,10) e com mães com ensino superior (77,9%; IC95% 76,56; 79,18). Unidades federativas com cobertura odontológica elevada apresentaram menor reporte de uso dos serviços, enquanto as com menor cobertura apresentaram maior proporção de ida ao dentista.

**Conclusão:**

Entre adolescentes de 13 a 15 anos observou-se prevalência de ida ao dentista no último ano de 66,7%; encontraram-se maiores prevalências de ida ao dentista entre os adolescentes do sexo feminino, autodeclarados brancos, com mães com maior escolaridade e com dor de dente autorreportada. Além disso, houve menor prevalência de ida ao dentista nos locais com maior cobertura odontológica.

**Aspectos éticos**

Esta pesquisa utilizou bancos de dados de domínio público e anonimizados.

## Introdução

Em 2022, aproximadamente um em cada sete brasileiros eram adolescentes com idade entre 10 e 19 anos (1). A adolescência é um período de fundamental importância no desenvolvimento humano, em que diferentes alterações físicas, psicológicas e emocionais ocorrem (2). Sendo um período transicional entre a infância e a fase adulta, é na adolescência que novos comportamentos em saúde são adotados, e, uma vez estabelecidos, repercutem diretamente sobre a vida adulta dos indivíduos (2-3). Particularmente, emergem novos comportamentos em saúde relacionados à dieta, atividade física, atividade sexual e uso de substâncias, como álcool e drogas (2-3). Muitos destes comportamentos podem contribuir para o estabelecimento ou agravamento de problemas de saúde bucal – como cárie dentária, gengivite, doença periodontal e má-oclusões, que podem interferir diretamente na função e estética, no desenvolvimento social e no desempenho escolar, além de contribuir para o absenteísmo escolar (4-7). Decorrentes destas condições, dor dentária e problemas estéticos também geram grandes prejuízos na qualidade de vida relacionada à saúde bucal, principalmente nos domínios de bem-estar social e emocional (8). 

No decorrer da adolescência ocorrem alterações significativas em características comportamentais em saúde bucal, muito relacionadas às mudanças nas influências sociais e à crescente autonomia e independência individual (2-3,9). Por exemplo, em estudo de coorte britânico observou-se um uso decrescente de serviços odontológicos no último ano entre a infância e o fim da adolescência, sendo a queda mais acentuada entre os 10 e 17 anos de idade (10). Diante dos diferentes padrões de uso de serviços de saúde entre diferentes períodos na adolescência, a Organização Mundial da Saúde preconiza o monitoramento da saúde do escolar por meio da vigilância de fatores de risco comportamentais e de proteção na faixa etária (11). Nessa perspectiva, o Ministério da Saúde tem incentivado os municípios a monitorarem a saúde dos escolares, buscando o planejamento e implementação de ações que promovam a saúde e que podem perdurar ao longo do ciclo vital (12).

Globalmente e no Brasil, embora jovens de 10 a 19 anos não estejam nas faixas-etárias de maior pico de prevalência e incidência dos agravos de saúde bucal, eles representam um período em que há um escalonamento progressivo dos índices de cárie dentária e de doença periodontal até a idade adulta (9,13). Desta forma, este período é uma janela de oportunidade relevante para intervenções que melhorem a saúde bucal dos indivíduos ao longo do ciclo vital. No Brasil, dados preliminares do Estudo Nacional de Saúde Bucal de 2022 revelaram que o número de jovens de 15 a 19 anos livres de cárie aumentou de 29,8% em 2010 para 33% em 2022 (14). Contudo, o componente de doença não-tratada aumentou dentro do índice (40% em 2010 versus 52,9% em 2022), o que sugere uma piora considerável na saúde bucal dos adolescentes brasileiros e uma maior necessidade de tratamento odontológico (14). Na mesma direção destes achados, estudo realizado com dados da Pesquisa Nacional de Saúde do Escolar (PeNSE) observou um aumento significativo da prevalência de dor dentária autorreportada de 18,8% em 2009 para 23,7% em 2015 (4).

Considerando a relevante carga de condições bucais em adolescentes, é essencial compreender os fatores sociodemográficos associados ao uso de serviços odontológicos por eles. Estudos apontam que jovens do sexo feminino (15-16), com mães de maior escolaridade e maior renda familiar têm maior probabilidade de terem ido ao dentista do que aqueles com pior condição socioeconômica (16-17). Além disso, iniquidades raciais e macrorregionais no uso dos serviços de saúde estão presentes; adolescentes de 13 a 17 anos e de cor de pele não-branca têm menor probabilidade de terem buscado os serviços de saúde no último ano (14) e, adolescentes (15 a 19 anos) da região Norte, Nordeste e Centro-Oeste têm maior probabilidade de nunca terem visitado o dentista (18); apesar disso, ambos os grupos utilizam proporcionalmente mais o serviço odontológico do Sistema Único de Saúde (SUS) (16, 18). Contudo, pouco se sabe sobre como estes fatores socioeconômicos impactam no uso de serviços odontológicos por adolescentes de 13 a 15 anos. Assim, o objetivo do presente estudo foi descrever a prevalência de ida ao dentista no último ano entre adolescentes de 13 a 15 anos participantes da PeNSE de 2019. 

## Métodos

### Delineamento do estudo

Este estudo transversal descritivo utilizou dados secundários da PeNSE do ano de 2019, que apresenta uma amostra representativa da população escolar brasileira de 13 a 17 anos. 

### Contexto e participantes

A PeNSE é um estudo realizado pelo Instituto Brasileiro de Geografia e Estatística juntamente com o Ministério da Saúde e Ministério da Educação, e possui quatro edições realizadas em 2009, 2012, 2015 e 2019 (19-20). A edição de 2019 foi realizada em escolas públicas e privadas, tendo abrangência nacional e dados disponibilizados para Brasil, Grandes Regiões, Unidades da Federação e Municípios da Capital (19). Os estudantes incluídos na pesquisa deveriam ter entre 13 e 17 anos e deveriam estar regularmente matriculados e cursando a escola, considerando o 6º ao 9º ano do ensino fundamental e do 1º ao 3º ano do ensino médio (19). Para a composição da amostra foi utilizada uma amostragem por conglomerados em dois estágios, considerando escolas o primeiro estágio e as turmas o segundo estágio (19). Informações adicionais sobre a PeNSE 2019 podem ser encontradas em publicação anterior (16). 

### Critérios de elegibilidade

No presente estudo, foram incluídos exclusivamente dados de adolescentes de 13 a 15 anos que apresentavam informações válidas para a variável dependente avaliada. A restrição da faixa etária está relacionada à ampliação da abrangência da amostra de escolares na edição de 2019 da PeNSE em comparação com as edições anteriores, passando a incluir representatividade por faixas etárias de 13 a 15 anos e de 16 a 17 anos (19). Segundo estimativas, a maioria dos adolescentes que frequentam a escola no Brasil está na faixa etária de 13 a 15 anos, correspondendo à amostra deste estudo (19).

### Variáveis de interesse

A variável dependente foi ida ao dentista no último ano, sendo mensurada pela pergunta “Nos últimos doze meses, quantas vezes você foi ao dentista?”. Esta pergunta poderia ser respondida com as seguintes opções de resposta: “Nenhuma vez nos últimos 12 meses”; “1 vez”; “2 vezes” ou “3 ou mais vezes”. Para fins de análise estatística, as respostas foram dicotomizadas em “Não” e “Sim”, sendo englobadas na categoria “sim” a ida ao dentista no último ano independentemente do número de vezes.

Como variáveis independentes foram utilizadas: sexo (Masculino e Feminino), raça/cor da pele (Branca, Preta, Parda, Amarela e Indígena), região brasileira (Norte, Nordeste, Centro-oeste, Sudeste e Sul), escolaridade materna (Sem escolaridade/Ensino Fundamental Incompleto, Ensino Fundamental Completo, Ensino Médio Completo e Ensino Superior Completo), e dor dentária nos últimos seis meses (Não e Sim). 

### Análise estatística

A análise dos dados foi realizada através do programa estatístico Stata 18.0 (*Stata Corp, College Station*, Texas, Estados Unidos da América). As frequências absolutas e relativas da variável dependente, ida ao dentista no último ano, em relação às variáveis independentes, foram verificadas através do teste Qui-quadrado de Pearson. Foi estabelecido um nível de confiança de 5% (p-valor<0,05) e os respectivos intervalos de confiança de 95% (IC95%). O comando svy para efeito de delineamento foi utilizado.

Ainda, uma análise ecológica complementar foi realizada utilizando dados secundários sobre a cobertura de saúde bucal. Esses dados foram coletados por uma pesquisadora em março de 2024, através do Sistema de Informações da Secretaria de Atenção Primária à Saúde, nos painéis de indicadores da atenção primária à saúde referentes a saúde bucal, disponíveis online em sisaps.saude.gov.br/painelsaps/saude-bucal. A relação entre a cobertura de saúde bucal da atenção primária do SUS - como variável contextual, e, a ida ao dentista no último ano dos estudantes foi apresentada graficamente. Os dados secundários foram coletados por unidade da federação, filtrados para o ano de 2018, ano mais próximo aos resultados da PeNSE 2019.

## Resultados

A PeNSE 2019 estimou em 124.898 escolares entre 13 e 17 anos. Destes alunos de 13 a 15 anos compuseram cerca de 66% da amostra, sendo está a proporção incluída neste estudo. 

Entre os adolescentes de 13 a 15 anos, foi observado que 50,8% eram do sexo feminino, 39,8% residiam na região sudeste, 43,2% autodeclarados pardos, e, 31,2% dos participantes cursava o 9º ano do ensino fundamental (Tabela 1). Para dor de dente, 21,4% reportaram ter sentido dor nos últimos seis meses (Tabela 1). Além disso, pode ser observada uma prevalência de uso de serviço odontológico entre adolescentes nos últimos 12 meses de 66,7% (Tabela 1). 

**Tabela 1:** Proporção (%) e intervalo de confiança de 95% (IC95%) da amostra segundo características sociodemográficas, uso de serviços odontológicos e dor dentária em escolares brasileiros de 13 a 15 anos, dados da Pesquisa Nacional de Saúde do Escolar. Brasil, 2019 (n=82.389)

	%	IC95%
Sexo		
Masculino	49,2	48,27; 50,04
Feminino	50,8	49,96; 51,73
**Raça/cor da pele**		
Branca	36,8	35,72; 37,82
Preta	12,8	12,15; 13,49
Parda	43,2	42,19; 44,28
Amarela	3,8	3,47; 4,08
Indígena	3,4	3,14; 3,76
**Região brasileira**		
Norte	10,3	9,13; 11,57
Nordeste	28,1	26,34; 29,94
Centro-oeste	8,3	7,65; 9,05
Sudeste	39,8	37,38; 42,28
Sul	13,5	12,35; 14,70
**Ano escolar**		
6º ano do Ensino Fundamental	0,2	0,17; 0,32
7º ano do Ensino Fundamental	11,1	9,79; 12,58
8º ano do Ensino Fundamental	29,9	26,99; 32,91
9º ano do Ensino Fundamental	31,2	27,76; 34,77
1º ano do Ensino Médio	23,4	20,54; 26,57
2º ano do Ensino Médio	4,1	3,27; 5,12
3º ano do Ensino Médio	0,1	0,04; 0,17
**Escolaridade materna**		
Sem escolaridade/Ensino Fundamental incompleto	26,8	25,78; 27,94
Ensino Fundamental completo	17,1	16,38; 17,80
Ensino Médio completo	32,4	31,56; 33,21
Ensino Superior completo	23,7	22,80; 24,62
**Dor de dente nos últimos 6 meses**		
Não	78,6	77,87; 79,31
Sim	21,4	20,69; 22,13
**Ida ao dentista no último ano**		
Não	33,3	32,34; 34,21
Sim	66,7	65,79; 67,66
**Frequência da ida ao dentista no último ano**		
Uma vez	22,0	21,31; 22,82
Duas vezes	22,6	21,82; 23,37
Três vezes ou mais	55,4	54,36; 56,35

Ainda, na tabela 2, observou-se que adolescentes do sexo feminino foram mais propensas a reportarem o uso do serviço odontológico, sendo representadas por 68,4% (IC95% 67,27; 69,49), com uma diferença de um pouco mais de 3 pontos percentuais quando comparado ao sexo masculino, representados por 65% (IC95% 63,83; 66,19). Os adolescentes autodeclarados brancos apresentaram maior prevalência de ida ao dentista no último ano (71,8%; IC95% 70,54; 72,92), uma diferença de 10 pontos percentuais em relação aos adolescentes autodeclarados pretos (61,9%; IC95% 59,76; 63,93). Também se notou que os adolescentes cujas mães não haviam frequentado a escola ou tinham ensino fundamental incompleto apresentaram uma prevalência de ida ao dentista de 61,6% (IC95% 59,89; 63,24), enquanto os adolescentes com mães que completaram o ensino superior apresentaram uma prevalência de 77,9% (IC95% 76,56; 79,18) para ida ao dentista no último ano (tabela 2). Adicionalmente, notou-se que 69,4% (IC95% 67,61; 71,10) dos adolescentes que confirmaram ter tido dor de dente nos últimos seis meses foram ao dentista no último ano (Tabela 2).

**Tabela 2:** Proporção (%) e intervalo de confiança de 95% (IC95%) da ida ao dentista no último ano segundo características sociodemográficas e dor dentária em escolares brasileiros de 13 a 15 anos, dados da Pesquisa Nacional de Saúde do Escolar. Brasil, 2019 (n=82.389)

	Ida ao dentista no último ano		
	Não (IC95%)	Sim (IC95%)	p-valor
Sexo			<0,001
Masculino	35,0 (33,81; 36,17)	65,0 (63,83; 66,19)	
Feminino	31,6 (30,51; 32,73)	68,4 (67,27; 69,49)	
**Raça/cor da pele**			<0,001
Branca	28,3 (27,08; 29,46)	71,7 (70,54; 72,92)	
Preta	38,1 (36,07; 40,24)	61,9 (59,76; 63,93)	
Parda	35,8 (34,43; 37,20)	64,2 (62,80; 65,57)	
Amarela	36,4 (32,66; 40,25)	63,6 (59,75; 67,34)	
Indígena	33,6 (30,21; 37,25)	66,4 (62,75; 69,79)	
**Escolaridade materna**			<0,001
Sem escolaridade/Ensino Fundamental incompleto	38,4 (36,76; 40,11)	61,6 (59,89; 63,24)	
Ensino Fundamental completo	34,9 (32,77; 37,20)	65,1 (62,80; 67,23)	
Ensino Médio completo	30,5 (29,24; 31,88)	69,5 (68,12; 70,76)	
Ensino Superior completo	22,1 (20,82; 23,44)	77,9 (76,56; 79,18)	
**Dor de dente nos últimos 6 meses**			<0,001
Não	34,5 (33,37; 35,61)	65,5 (64,39; 66,63)	
Sim	30,6 (28,90; 32,39)	69,4 (67,61; 71,10)	

A Figura 1 ilustra a sobreposição da cobertura odontológica na atenção básica e proporção reportada de ida ao dentista no último ano entre os estudantes por unidade da federação. A maior cobertura foi observada para o estado do Piauí, com 97,5% da população assistida, seguido da Paraíba, com 92,1% e Tocantins com 88,3% (Figura 1). Em contrapartida, o Distrito Federal apresentou apenas 27,9% de cobertura para a população (Figura 1). Para a proporção de ida ao dentista no último ano separadamente, o estado de Santa Catarina apresentou o reporte de 75% dos adolescentes, seguido do Rio Grande do Sul (71,6%), São Paulo (70,9%) e Paraná (70,6%); observou-se a menor proporção no estado do Amapá, com 55,3%. Desta forma, pode ser destacado que unidades da federação com cobertura elevada, como as da região Norte e Nordeste tiveram a sobreposição com um menor reporte de uso dos serviços pelos adolescentes, enquanto unidades federativas com menor cobertura apresentaram maior proporção de ida dos estudantes ao dentista no último ano. 

**Figura 1:** Sobreposição da proporção de ida ao dentista no último ano com dados da Pesquisa Nacional de Saúde do Escolar 2019, em relação à cobertura odontológica da atenção básica por Unidade Federativa com dados Sistema de Informações da Secretaria de Atenção Primária à Saúde no Brasil, 2018

## Discussão

Dentre os achados deste estudo pode ser destacada a alta prevalência do uso do serviço odontológico entre adolescentes de 13 a 15 anos participantes da PeNSE 2019. Ainda, notou-se um maior relato de uso do serviço odontológico por adolescentes do sexo feminino, raça/cor de pele autorreportada branca, com mães com maior escolaridade e que sentiram dor de dente nos últimos seis meses em comparação às contrapartes. Adicionalmente, deve ser destacada a sobreposição gráfica inversa entre a cobertura de saúde bucal e a proporção de ida ao dentista no último ano por unidades federativas.

Apesar da prevalência de uso de serviço odontológico no último ano poder ser considerada elevada em comparação a outros países de média renda (21-22), ocorreu uma redução em comparação aos dados da edição anterior da PeNSE, em 2015 (4,19). Essa redução não só sugere uma menor busca por serviços odontológicos, mas também pode resultar no surgimento de novos problemas de saúde bucal ou na progressão de doenças bucais pré-existentes (23). Além disso, observou-se que o maior relato de dor dentária nos últimos seis meses esteve associado a um maior uso do serviço odontológico no último ano, o que reforça um padrão já observado de uso dos serviços de saúde bucal majoritariamente para fins curativos (16-17).

Corroborando achados do presente trabalho, uma pesquisa conduzida com dados da PeNSE de 2015 havia observado um maior reporte do uso dos serviços de saúde por estudantes do sexo feminino (16).

Ainda, dados nacionais apontaram que mulheres tendem a procurar mais os serviços de saúde (24), este maior uso do serviço pode ser atribuído à preocupação com questões e cuidados em saúde, aumentando as possibilidades de diagnóstico e prevenção. A observação de uma diferença significativa na frequência de consultas odontológicas entre jovens brancos e não brancos, reforça que este estudo também reitera o impacto das iniquidades raciais na utilização dos serviços de saúde. Já foi constatado que indivíduos negros possuem maior probabilidade de realizar um tratamento odontológico irregular ou, até mesmo, nunca terem consultado o dentista (25). Esta discrepância também foi encontrada em um estudo com escolares adolescentes brasileiros que observou que jovens pretos e pardos sofrem mais com a dor dentária em comparação aos brancos (26). Desde cedo, pessoas negras precisam lidar com piores condições de vida, que repercutem no acesso e no uso de serviços de saúde, além de lidarem com o racismo estrutural, institucional e interpessoal que impacta quantitativamente o cuidado em saúde recebido (25,27).

Ademais, nota-se que a ida ao dentista no último ano tende a aumentar conforme cresce o nível de escolaridade materna, com uma diferença na prevalência de ida ao dentista de 16 pontos percentuais entre aqueles com mães de menor escolaridade e as mais escolarizadas. Isso pode refletir o menor conhecimento sobre cuidados com a saúde bucal, dificuldades no acesso à informação sobre os serviços de saúde e, principalmente, sobre a presença de dentistas em unidades básicas de saúde (28). Faz-se necessário vincular a questão da renda familiar, uma vez que o maior nível de escolaridade é associado a um maior poder econômico, de forma que grupos familiares de maior poder aquisitivo tendem a acessar mais serviços de saúde, sendo na maioria das vezes, em consultórios privados (29-30). Uma menor frequência de ida ao dentista no grupo dos adolescentes com mães menos escolarizadas pode estar relacionada ao horário de funcionamento dos serviços públicos de saúde, pois normalmente coincidem com o horário escolar ou até mesmo, caso o estudante trabalhe em meio período, com seu horário de trabalho ou também de seus pais (4,31).

A relação inversa entre a proporção de cobertura de saúde bucal na atenção básica e a proporção de ida ao dentista no último ano, observada no presente estudo, poderia ser explicada por dois fatores principais. Regiões mais afastadas do país, principalmente retratadas pela região norte, apresentam dificuldades quanto a espaços físicos, deslocamento de equipes e fixação de profissionais, porém sua população ainda tem como principal referência os serviços públicos de saúde (32-33). Entretanto, uma vez que os estados com alta cobertura da atenção básica são também aqueles com uma maior concentração de renda, isso reforça a ideia de que as consultas sendo realizadas majoritariamente em serviços privados ou por convênios, ou baixa confiança nos serviços prestados pelo Sistema Único de Saúde (18,34). 

Dentre os pontos fortes deste estudo pode-se citar a utilização de uma amostra representativa para o universo de escolares brasileiros entre 13 e 15 anos, o que garante a generalização dos nossos achados. Também, reforça-se o uso de dados da última edição da PeNSE, o que atualiza a literatura já disponível sobre o tema e o foco específico no estrato de jovens de 13 a 15 anos. Entretanto, algumas limitações devem ser destacadas. É necessário ressaltar que mais da metade dos adolescentes de 13 a 15 anos que foram ao dentista nos últimos 12 meses alegaram terem ido três vezes ou mais. Como o questionário aplicado aos alunos não descartava a visita ao dentista para tratamento ortodôntico, procedimento muito comum na idade analisada neste estudo, essa prevalência pode ter sido influenciada pela adesão e continuidade a este tipo de tratamento (23). Assim, cita-se como limitação relevante desta pesquisa o fato de que os dados não permitem distinguir atendimentos por motivos preventivos de curativos, dificultando uma análise mais detalhada do motivo do uso do serviço odontológico nessa população. Ademais, como apenas adolescentes que estão devidamente matriculados e frequentes na escola são elegíveis para a PeNSE, os resultados aqui encontrados não são generalizáveis para jovens fora da escola e, consequentemente, em maior vulnerabilidade. 

Em conclusão, os achados deste estudo indicam que o uso dos serviços odontológicos por adolescentes de 13 a 15 anos no Brasil pode estar associado a desigualdades socioeconômicas e demográficas, evidenciando iniquidades no acesso ao serviço. Além disso, a relação inversa observada entre a cobertura odontológica da atenção básica e a ida ao dentista sugere a necessidade de investigar possíveis barreiras na utilização desses serviços. Esses resultados reforçam a importância de políticas públicas que garantam um acesso mais equitativo à assistência odontológica desde o início da adolescência, contribuindo para a redução das desigualdades em saúde bucal.

## Data Availability

Os dados utilizados neste artigo estão disponíveis em: https://osf.io/swn8y/?view_only=cf392fd83fc54493b836f8c463063f57
